# Genetic Evaluation and Selection of Growth Traits of *Pinus kesiya* var. *langbianensis* Half-Sib Families

**DOI:** 10.3390/plants15132035

**Published:** 2026-06-30

**Authors:** Xiaoliang Che, Qiang Han, Xia Zhao, Guihua Huang, Xianbang Wang, Jianmin Xu, Yong Yang, Liyong Chen, Yundong Zhang

**Affiliations:** 1 Research Institute of Tropical Forestry, Chinese Academy of Forestry, Guangzhou 510520, China; chexl970128@126.com (X.C.); hanqiang1988@caf.ac.cn (Q.H.); ritfzx@caf.ac.cn (X.Z.); ritfhuanggh@caf.ac.cn (G.H.); wangxb@caf.ac.cn (X.W.); 2 Pu’er Weiguo State-Owned Forestry Bureau, Pu’er 665199, China; 3 Pu’er Forestry Industry Group Co., Ltd., Pu’er 665000, China

**Keywords:** half-sib progeny trial, variance component estimation, forward and backward selection, Bayesian threshold model, tropical conifer

## Abstract

*Pinus kesiya* var. *langbianensis* is a major commercial conifer in subtropical Yunnan, China, yet systematic genetic evaluation of its breeding populations remains scarce. We estimated genetic parameters for growth and stem form (SF) traits to support advanced-generation selection. In a progeny trial of 113 open-pollinated half-sib families established in 2013 at Pu’er, 2201 surviving trees were assessed at age 11 for height, diameter at breast height (DBH), stem volume, height to crown base (HCB), crown width, and an ordinal SF score. Variance components and breeding values (BVs) were estimated using linear mixed models, with a Bayesian threshold model as a sensitivity analysis for ordinal data. Family-mean heritabilities showed a gradient across growth traits (height 0.14, DBH 0.29, and volume 0.24). Stem volume had the highest genetic coefficient of variation (18.85%), the optimal selection target, whereas HCB lacked detectable genetic variance and SF was weakly controlled (0.09). Phenotypic and BV rankings diverged: although Yunjing Seed Orchard showed the highest phenotypic means, best linear unbiased prediction identified Jinggu Seed Orchard as contributing nine of the top ten families and 26 of the 35 elite individuals. A combined backward–forward strategy, ranking materials by volume BVs under a phenotypic threshold (SF ≥ 7), yielded predicted volume gains of 14.35% (family) and 15.01% (individual), providing a basis for second-cycle breeding pending multi-environment validation.

## 1. Introduction

Coniferous plantations supply a substantial share of the world’s industrial roundwood, and they are increasingly expected to contribute to broader terrestrial ecosystem goals alongside timber production [1]. The genus *Pinus*, the largest and most economically valuable group of conifers in the Northern Hemisphere [2], has been the focus of systematic genetic improvement for more than half a century. Long-term recurrent selection in loblolly pine (*Pinus taeda* L.), radiata pine (*P. radiata* D. Don), and slash pine (*P. elliottii* Engelm.) has routinely delivered volume gains of 20–35% per breeding cycle [3,4,5]. These gains rest on a well-established quantitative-genetic framework: variance components and inter-trait genetic correlations are estimated from progeny trials, breeding values are predicted via best linear unbiased prediction (BLUP), and multi-trait selection indices are used to reconcile competing breeding objectives [6].

Open-pollinated half-sib family trials remain the most widely used platform in the early phases of conifer breeding. Their appeal lies in a simple design that permits the simultaneous estimation of family performance, additive genetic variance, and breeding values [7,8]. For growth traits such as diameter at breast height (DBH), total height, and stem volume, the level of additive genetic control commonly observed in *Pinus* has consistently justified the use of half-sib progeny trials as the primary platform for parameter estimation and family selection [4,5,9]. Stem form traits, including straightness and branch habit, are under weaker genetic control, but many *Pinus* populations still show enough additive variation to justify their use as secondary selection criteria [10]. A persistent obstacle in these programs is the unfavorable genetic correlation between volume growth and wood quality and between growth rate and stem form; when single-trait selection is applied, such correlations can erode the economic value of the selection response [4,11]. Reliable genetic parameter estimates are therefore essential for deciding which traits warrant inclusion in a selection program and which are better addressed through other means [6].

Simao pine (*Pinus kesiya* var. *langbianensis*) is an ecologically and economically important native conifer in southwestern China. It accounts for roughly 11% of the forest area and more than 90% of the gum-turpentine output of Yunnan Province [12,13]. Despite its economic importance, Simao pine breeding has progressed slowly compared to other major Chinese conifers (e.g., *Cunninghamia lanceolata* and *P*. *massoniana*), and the species remains largely confined to first-generation selection [13]. Few quantitative genetic analyses have been published for Simao pine, and those that exist share two fundamental limitations. Most previous evaluations rely on small family sample sizes [13,14] and focus exclusively on growth, leaving a notable gap in systematic genetic estimates for stem form and its correlation with growth traits [13,14,15]. Although studies on the congener *P. kesiya* in Malawi report moderate-to-high heritabilities [9,11], the strict population- and environment-specificity of genetic parameters means these estimates cannot simply be applied to Simao pine in southwestern China [4,6].

The present study addresses this gap using an 11-year-old open-pollinated progeny trial of 113 half-sib families of *P. kesiya* var. *langbianensis*. The selected superior individuals are intended to serve two complementary purposes: parents for a second-generation breeding population and ortets for vegetative propagation (grafting and cutting) in the production deployment of Simao pine plantations. The specific objectives were to: (i) characterize phenotypic variation among seed sources and families for growth traits and stem form; (ii) estimate family-mean heritabilities and genetic correlations for these traits using linear mixed models fitted by restricted maximum likelihood (REML); and (iii) identify superior families and individuals through BLUP breeding values for stem volume, the primary breeding objective, while applying a stem form criterion at the individual level to ensure that candidates selected for vegetative propagation meet the quality requirements of operational plantation forestry.

## 2. Results

### 2.1. Phenotypic Variations Among Seed Sources and Families

**Figure 1:**
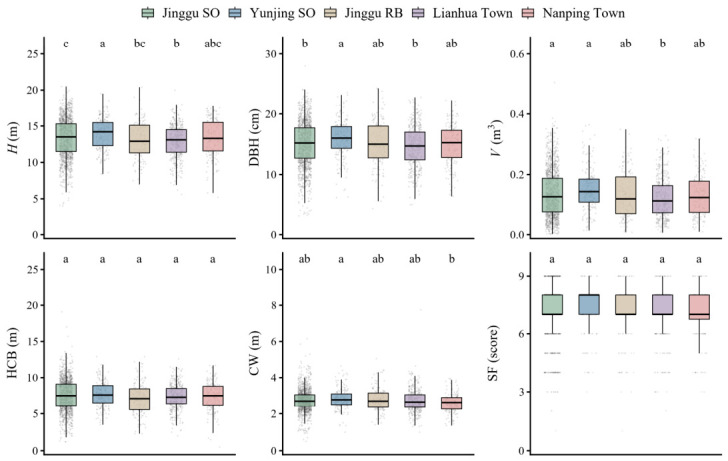
Phenotypic variation in growth and stem form traits among five seed sources of *Pinus kesiya* var. *langbianensis*. Box plots show the median, interquartile range, and min–max whiskers, with jittered individual data points overlaid. Different lowercase letters indicate significant differences among seed sources (*p* < 0.05). SO, Seed Orchard; RB, Resin Base.

Significant differences among the five seed sources were detected for height (H), diameter at breast height (DBH), individual stem volume (V), and crown width (CW) (*p* < 0.05; Figure 1), whereas height to crown base (HCB) and stem form (SF) did not differ significantly among sources. Yunjing Seed Orchard (Source 2) ranked highest for H, DBH, and CW and, together with Jinggu Seed Orchard (Source 1), showed the highest V. Its mean H, DBH, and V exceeded those of Lianhua Town by 8.1%, 8.5%, and 22.8%, respectively. Lianhua Town and Nanping Town generally occupied the lower rankings for H, V, and CW. Among-source differentiation was therefore most pronounced for stem volume, while tree height to crown base and stem form showed no significant variation among the seed sources studied.

At the family level, significant differences were detected among the 113 half-sib families for all traits (*p* < 0.01, Table 1). All traits means at age 11 were 13.29 m for H, 15.06 cm for DBH, 0.140 m^3^ for V, 7.48 m for HCB, 2.73 m for CW, and 7.2 score for SF; family-mean ranges and phenotypic coefficients of variation are summarized in Table 2. Stem volume exhibited the highest phenotypic coefficient of variation (PCV = 47.56%), while DBH, HCB, and CW each exceeded 20%; H and SF showed the lowest variability. There were highly significant differences for all traits among Block effects and Family × Block interactions (*p* < 0.01), indicating substantial within-site environmental heterogeneity and rank instability across blocks.

### 2.2. Genetic Variance Components and Heritability

Genetic analysis revealed substantial variation in the degree of genetic control among the six traits (Table 2). DBH and V exhibited moderate heritability, with family mean heritability ($h_{f}^{2}$) of 0.29 and 0.24 and individual narrow-sense heritability ($h_{s}^{2}$) of 0.12 and 0.11, respectively. Stem volume had the highest genetic coefficient of variation (GCV_A_ = 18.85%). In contrast, HCB displayed negligible genetic variance ($\sigma_{A}^{2}\approx 0$, $h_{s}^{2}\approx 0$) and was therefore excluded from subsequent correlation and selection analyses. *SF* heritability was also low ($h_{f}^{2}$ = 0.09, $h_{s}^{2}$ = 0.04); GCVA was not computed for SF because the Blom transformation alters the original scale and mean.

### 2.3. Phenotypic and Genetic Correlations

**Figure 2:**
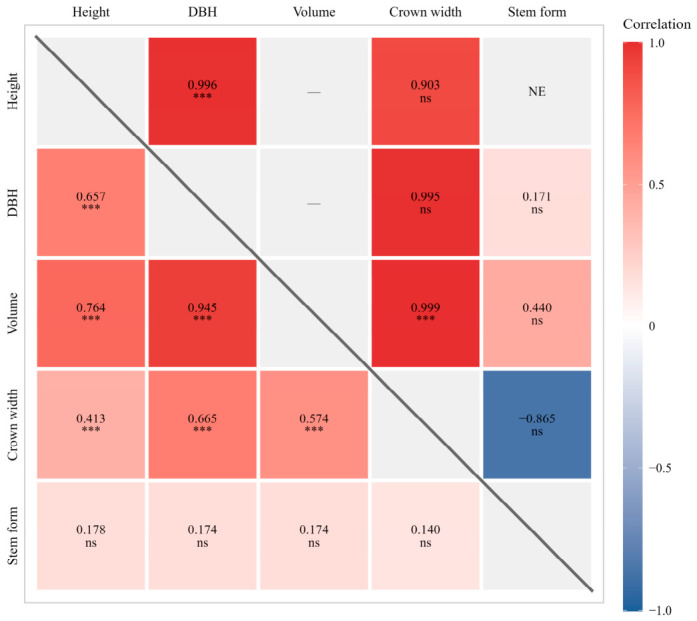
Heatmap of phenotypic and genetic correlations among six traits in *Pinus kesiya* var. *langbianensis*. The lower triangle (below the diagonal) displays phenotypic correlation coefficients ($r_{p}$), while the upper triangle (above the diagonal) displays additive genetic correlation coefficients ($r_{g}$). The color gradient represents the strength and direction of correlations, ranging from deep blue (strong negative correlation) to deep red (strong positive correlation). Significance levels: *** = *p* < 0.001; ns, not significant; NE, not estimable.

Correlation analyses were conducted on five traits (H, DBH, V, CW, SF) following the exclusion of HCB (Figure 2). For growth traits, genetic correlations ($r_{g}$) generally exceeded their corresponding phenotypic correlations ($r_{p}$) in magnitude, indicating that these associations are primarily driven by pleiotropic genetic effects. Phenotypic correlations among height, DBH, volume, and crown width were positive, moderate to strong ($r_{p}$= 0.413–0.945), and highly significant (*p* < 0.001). At the genetic level, height and DBH showed a nearly perfect correlation ($r_{g}$ = 0.996, *p* < 0.001), as did volume and crown width ($r_{g}$ = 0.999, *p* < 0.001). Genetic correlations between volume and the traits from which it is derived (height and DBH) were omitted to avoid structural multicollinearity.

In contrast, the correlations involving SF behaved anomalously. Phenotypic correlations between SF and growth traits were uniformly weak and non-significant ($r_{p}$ = 0.140–0.178), and the estimated genetic correlations included a non-estimable value between SF and height as well as a biologically implausible estimate of −0.865 for crown width with SF (non-significant).

### 2.4. Sensitivity Analysis for Stem Form

The Bayesian threshold mixed model, fitted directly to the original ordinal SF scores, yielded a liability-scale heritability of 0.119 (95% highest posterior density HPD: 0.000–0.261), consistent with the low estimate from the Blom-transformed LMM approach ($h_{s}^{2}$ = 0.04; Supplementary Table S1). Bivariate threshold–linear models further showed that genetic correlations between SF and the four primary selection traits (H, DBH, V, CW) were all statistically non-significant, with 95% HPD intervals spanning zero (Supplementary Table S2). All models achieved satisfactory Markov Chain Monte Carlo (MCMC) convergence (effective sample size > 1000, |Geweke z| < 2) (Supplementary Table S3). The anomalous LMM genetic correlation estimates for SF can therefore be attributed to insufficient additive genetic variance rather than genuine biological signals.

### 2.5. Selection of Superior Families and Elite Individuals

Building on the genetic parameter estimates and sensitivity analyses, the selection strategy was optimized to maximize wood production while ensuring acceptable stem quality. Because height to crown base (HCB) lacked additive genetic variance and stem form (SF) exhibited extremely low heritability, these traits were excluded from genetic ranking. Instead, a two-stage independent culling strategy was implemented: candidates were ranked strictly by their estimated breeding values (BVs) for stem volume, while a phenotypic safeguard was applied for stem form.

At the family level, the top 10 superior families were identified based on their mean BVs for stem volume (Table 3). Notably, nine of these top-performing lineages originated from Seed source 1, highlighting the superior genetic potential of this specific provenance. The selected family cohort achieved a mean BV of 0.0194, which corresponds to a predicted genetic gain of 14.35% for stem volume relative to the base population.

At the individual level, 35 elite trees were selected across the test plantation based on their individual stem volume BVs (Table 4). To safeguard timber quality, a strict independent culling threshold was enforced, rejecting any candidate with an SF score below 7. Furthermore, in order to manage coancestry and to preserve genetic diversity for the advanced-generation seed orchard, selections were restricted to a maximum of two individuals per top-10 family and one individual per remaining superior family. This elite individual cohort yielded a mean BV of 0.0203, generating a genetic gain of 15.01% for stem volume. The marginal improvement in gain from individual selection compared to family selection (15.01% vs. 14.35%) corroborates the low narrow-sense heritability of the trait, indicating that the bulk of the exploitable additive genetic variance resides among, rather than within, families. Nonetheless, these 35 elite individuals constitute the optimal foundational material for next-generation controlled crossing and clonal deployment.

## 3. Discussion

### 3.1. Genetic Control Gradients in Growth Traits

The three growth traits in this 11-year-old *P*. *kesiya* var. *langbianensis* population showed a clear gradient of additive genetic control, with tree height returning the lowest family-mean heritability ($h_{f}^{2}$ = 0.14) and DBH and stem volume the highest (0.29 and 0.24, respectively). Within *Pinus* more broadly, narrow-sense heritabilities for growth traits typically fall in the range of 0.15 to 0.35, and family-mean heritabilities frequently exceed 0.50 [4,9], and the gradient observed here has direct parallels in other species at comparable or older ages: cross-sectional and longitudinal data in *P. koraiensis* [16], *P. elliottii* [5], *P. massoniana* [17], and *P. taeda* [18] consistently show tree height heritability as more variable and often lower than DBH heritability, with the latter remaining stable or even increasing toward mid-rotation. The biological basis for this pattern lies in the heightened sensitivity of tree height to stand-level competition, as apical growth becomes constrained by local light microenvironments once the canopy closes. Lee et al. [16] reported that, in *P. koraiensis*, tree-height heritability was relatively high before age 23 and declined thereafter, whereas DBH heritability increased with age and peaked around age 30. They attributed the age-related decline in height heritability to increasing environmental noise, including inter-tree competition, which progressively weakens the detectable family-level genetic signal for height. The present Simao pine trial, assessed at age 11 under relatively dense planting (2.5 m × 3.0 m), has likely reached this competitive threshold, and the lower tree height heritability observed here is consistent with this developmental context.

Within this gradient, stem volume emerges as the trait offering the largest genetic improvement potential despite not having the highest heritability. Volume integrates additive variation in both height and diameter multiplicatively, producing an additive genetic coefficient of variation of 18.85% in this population—approximately twice the typical values reported for tree height and DBH in pine breeding studies [19,20]. The predicted gains of 14.35% and 15.01% at the family and individual levels, respectively, confirm that this proportional advantage translates into observable selection response. These conclusions are conditional on the single-site, age-11 measurement; mid-rotation re-evaluation will be needed to verify that volume retains its advantage at commercial maturity.

### 3.2. Environmental Regulation of Form-Related Traits

The two form-related traits assessed here showed weak genetic control: HCB had no detectable additive variance ($\sigma_{A}^{2}$ ≈ 0), and SF returned a low family-mean heritability of 0.09. The genetic correlations involving SF were similarly unstable—non-estimable with tree height and implausibly negative (−0.865) with crown width—reflecting the small additive component for this trait. The Bayesian threshold model fitted to the original ordinal scores confirmed these unstable estimates as statistical artifacts rather than biological signals. Comparable patterns of weak additive control for stem form are recurrent in tropical pine systems. Stem-straightness heritabilities below 0.18 in *P. elliottii* × *P. caribaea* [21,22] and in *P. pinaster* [23], contrasted with 0.23 to 0.55 in *P. radiata* [24], indicate that the present finding follows a broader tropical-subtropical pattern.

The biological basis for this pattern lies in the dependence of form-related traits on stand-level processes that are largely external to the individual genotype. Self-pruning—the mechanism that determines HCB—proceeds through branch suppression and decay driven by light availability and crown depth [25], making the live-crown position an emergent property of local light environments shaped by spacing, mortality, and microsite variation. Stem form is shaped by similar competitive dynamics, although less acutely. In *P. taeda*, competition reduced DBH heritable variance to under 43% of its competition-free level, while stem form was comparatively robust [26]. The low SF heritability observed here is therefore unlikely to be an artifact of competition-induced noise.

Gains in stem form and crown structure may rely more on silvicultural management than on genetic selection alone. Spacing, thinning intensity, and pruning all influence form development, though with thresholds: in *P. patula*, only very low planting densities reduced stem straightness substantially [27]. Silviculture therefore complements rather than replaces a coordinated strategy in which growth traits are selected genetically and form traits managed environmentally. Beyond the within-stand environmental factors, a partial contribution from trans-generational epigenetic effects to the observed source-level differences cannot be excluded, although the broadly similar climatic and edaphic conditions across the maternal sites in southern Yunnan suggest this contribution is unlikely to be a major driver of the patterns observed.

### 3.3. Selection Strategy from Phenotypic to Breeding Values

Two patterns in the elite cohort merit consideration. While the discrepancy between Yunjing’s high phenotypic mean and its modest BLUP representation relative to Jinggu is primarily a sampling artifact (10 Yunjing families vs. 68 from Jinggu), the substantive signal is the systematic divergence between managed sources and unselected natural stands. The three managed sources (seed orchards and resin base) contributed to all top ten families and 32 of 35 elite individuals. This outcome serves as a testament to the foundational plus-tree selection work in Yunnan. The 14.35% and 15.01% predicted volume gains obtained here reflect the operational realization of the high expected gains previously reported for Simao pine [15]. Evidently, prior parent-tree selection successfully elevated the mean breeding values of managed sources, with the Jinggu Resin Base’s intermediate ranking confirming that such selection benefits are strictly trait-specific.

However, sustained selection in clonal orchards risks reducing effective population sizes and depleting low-frequency alleles [28,29]. The natural-stand-derived families therefore retain a critical complementary role. By supplying high-breeding-value (high-BV) individuals not yet integrated into the formal orchard system, these sources offer a targeted mechanism to broaden the second-cycle genetic base without compromising overall merit. This aligns with frameworks for integrating gene resource populations [30] and strategies advocating continuous plus-tree infusion from diverse provenances to secure long-term gain [31].

The applied selection strategy synthesizes backward and forward selection. Family-level BLUP rankings provide backward-selection criteria to identify superior parent trees for advanced-generation seed orchards [32], yielding volume gains consistent with recent *P. taeda* evaluations [33]. Concurrently, individual-level BLUPs enable forward selection of 35 elite genotypes for direct deployment in the second-cycle breeding population [34,35]. To satisfy vegetative propagation requirements, a phenotypic threshold (SF ≥ 7) was superimposed [6]. With 28 of the 35 elite individuals scoring 8 or 9, this rule validated rather than constrained candidate availability. Multi-environment trials remain necessary to verify the generalizability of these rankings beyond the Pu’er site.

## 4. Materials and Methods

### 4.1. Study Site Description

**Figure 3:**
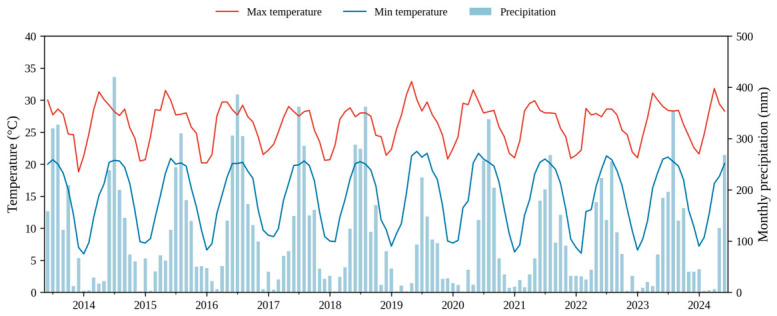
Monthly maximum temperature, minimum temperature, and precipitation at the trial site from June 2013 to June 2024, based on the TerraClimate dataset (~4 km resolution). Lines show monthly maximum and minimum temperatures; bars show monthly precipitation.

The progeny trial was established in Xiaoheijiang Forest Park (23°05′ N, 101°02′ E; 1200–1400 m a.s.l.), Ning’er County, Yunnan Province, China. The area has a subtropical highland monsoon climate. According to long-term records of the Ning’er County Meteorological Bureau, the mean annual temperature is 18.2 °C and the mean annual precipitation is 1398 mm, with approximately 88% of the rainfall concentrated in the May–October wet season and a mean annual relative humidity of about 78%. Monthly temperature and precipitation over the entire experimental period (June 2013 to June 2024) were derived from the TerraClimate dataset [36] (Figure 3), illustrating the pronounced monsoonal seasonality and the interannual variation in rainfall.

At each of the five evenly distributed sampling points across the trial site, approximately 1 kg of soil was collected from the 0–60 cm layer and thoroughly mixed to form a composite sample, which was then analyzed following the Chinese Forestry Industry Standard [37,38,39,40,41] (Table 5). The soils are strongly acidic (pH 4.9 ± 0.09) with a clay loam texture (clay 35%, silt 28%, sand 37%), characterized by low total phosphorus (0.19 ± 0.01 g kg^−1^) and very low available phosphorus (0.53 ± 0.08 mg kg^−1^), a soil organic carbon content of 6.12 ± 1.77 g kg^−1^, and a total nitrogen content of 0.62 g kg^−1^ (C/N ratio 9.9 ± 0.53). These acidic, phosphorus-deficient red soils are typical of the natural habitat of Simao pine (*P. kesiya* var. *langbianensis*) in southern Yunnan.

The trial is located within a national forest park under regular management. During the experimental period (2013–2024), no fire, pest outbreak, or disease epidemic was recorded at the site.

### 4.2. Plant Materials and Experimental Design

**Figure 4:**
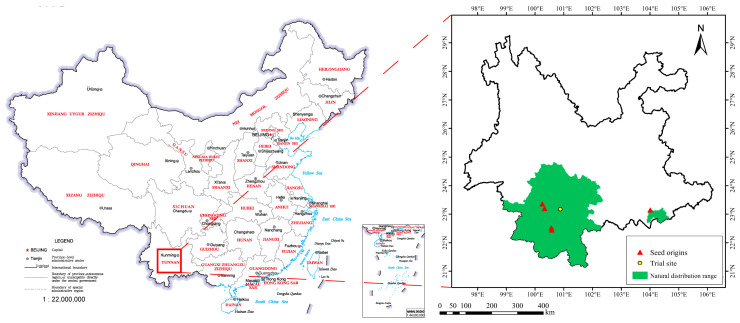
Geographical distribution of the *Pinus kesiya* var. *langbianensis* progeny trial and five seed sources. The green shaded area indicates the natural distribution range of Simao pine in Yunnan Province; the yellow dot marks the location of the experimental site in Ning’er County; the red triangles denote the five sampled seed sources.

The plant materials consisted of 113 open-pollinated half-sib families of Simao pine (*P. kesiya* var. *langbianensis*) together with one commercial check lot (CK). The families were collected from five seed sources spanning the natural distribution range of the species in southern Yunnan (Figure 4, Table 6).

The field trial was established in June 2013 following a Randomized Complete Block Design (RCBD) with four blocks. Within each block, families were arranged in single-row plots of five trees at a spacing of 2.5 m × 3.0 m. Each half-sib family was nominally represented by four five-tree plots (20 trees per family); due to higher seedling availability for certain families during the 2013 planting operation, some families had one or more additional five-tree plots, with initial planting numbers per family ranging from 20 to 35 trees. The detailed sampling structure of the 113 half-sib families across the five seed sources at age 11 is summarised in Supplementary Table S4; the total of 2201 surviving trees fully accounts for all individuals included in the variance component, heritability, and BLUP analyses reported in this study. Two continuous buffer rows of Simao pine were planted around the experimental area to mitigate edge effects.

### 4.3. Data Collection

A comprehensive field evaluation and survival census were conducted 11 years post-planting. Six traits were measured on all surviving trees: Total tree height (H, m) and height to crown base (HCB, m) were measured with a Haglöf Vertex IV hypsometer (0.1 m precision) (Haglöf Sweden AB, Långsele, Sweden); diameter at breast height (DBH, cm) was measured at 1.3 m above ground with a diameter tape (0.1 cm precision); crown width (CW, m) was the average of two perpendicular (east–west and north–south) crown diameters measured with a tape (0.1 m precision); and stem form (SF, score) was visually assessed on a 1–9 scale, where 1 indicates an unacceptable, severely crooked stem and 9 indicates a perfectly straight stem, following the standard assessment guidelines for international *P. kesiya* perveance trials [42]. Individual tree stem volume (V, m^3^) was calculated using Equation (1).

(1) $$V=0.000051577714\times {DBH}^{1.985218}\times H^{0.92035096}$$

### 4.4. Statistical Analysis

#### 4.4.1. Data Preprocessing and Seed Origin Variations

For the five continuous traits (H, HCB, DBH, V, and CW), normality and homogeneity of variances were assessed by the Shapiro–Wilk test and Levene’s test, respectively. Seed source means were compared using one-way ANOVA followed by Tukey’s HSD test for traits meeting the assumption of homogeneity of variances or Welch’s ANOVA followed by the Games–Howell test for heteroscedastic traits. Stem form (SF), an ordinal score, was analyzed using the Kruskal–Wallis test followed by Dunn’s test with Benjamini–Hochberg correction for multiple comparisons.

The stem form (SF), being an ordinal 1–9 with a strongly right-skewed distribution, was not normalized by square-root transformation. Blom normal scores [43] were therefore calculated using Equation (2) and used for all subsequent linear mixed model analyses of SF:

(2) $$\mathrm{Blom}(r)=\Phi^{-1}\times [(r-3/8)/(n+1/4)]$$

where *r* is the rank of the observation, *n* is the total sample size, and Φ^−1^ is the inverse of the standard normal cumulative distribution function. All preprocessing and seed-source-level analyses were performed in R v4.4.2 [44].

#### 4.4.2. Genetic Parameters and BLUP Evaluation

Variance components were estimated by restricted maximum likelihood (REML), and breeding values were predicted using Best Linear Unbiased Prediction (BLUP) with the ASReml-R package (v4.2) [45]. Two complementary linear mixed models were fitted. The family model (3) was used to estimate variance components and family-level breeding values, which provided the basis for family ranking at Stage 1 of the selection procedure. The individual (animal) model (4), which incorporates the numerator relationship matrix derived from the half-sib pedigree, was used to predict individual breeding values for Stage 2 selection.

(3) $$Y_{ijk}=\mu +B_{i}+F_{j}+BF_{ij}+\varepsilon_{ijk}$$

where $Y_{ijk}$ is the phenotypic observation, μ is the overall mean; $B_{i}$ is the fixed effect, $F_{j}$ is the random effect, ${BF}_{ij}$ is the random family-by-block interaction effect, and $\varepsilon_{ijk}$ is the random residual.

(4) y=Xβ+Zα+e

where *y* is the vector of phenotypic observations, β is the vector of fixed effects (overall mean and block), α is the vector of random additive genetic effects with α ~ N (0, A $\sigma_{A}^{2}$), in which A is the numerator relationship matrix, *X* and *Z* are the incidence matrices for the fixed and random effects, respectively, and *e* is the vector of random residuals.

Variance components extracted from the mixed model were used to calculate narrow-sense individual heritability ($h_{s}^{2}$), family mean heritability ($h_{f}^{2}$), and the genetic coefficient of variation (GCV_A_) according to Equations (5), (6), and (7), respectively [46]:

(5) $$h_{s}^{2}=\frac{4\sigma_{f}^{2}}{\sigma_{f}^{2}+\sigma_{f^{*}b}^{2}+\sigma_{\varepsilon }^{2}}$$

(6) $$h_{f}^{2}=\frac{\sigma_{f}^{2}}{\sigma_{f}^{2}+\frac{\sigma_{f^{*}b}^{2}}{b}+\frac{\sigma_{\varepsilon }^{2}}{n_{h}b}}$$

(7) $$GCV_{A}=\frac{\sigma_{A}}{\bar{X}}\times 100\%$$

where $\sigma_{A}^{2}=4\sigma_{f}^{2}$ represents the additive genetic variance (assuming half-sib families), $\sigma_{f}^{2}$ is the family variance, $\sigma_{fb}^{2}$ is the family-by-block interaction variance, $\sigma_{\varepsilon }^{2}$ is the residual variance, b is the number of blocks, *n* is the harmonic mean of trees per plot, and $\bar{X}$ is the phenotypic mean. Phenotypic and genetic correlations between pairs of traits were estimated from the corresponding phenotypic and additive genetic (co)variance components using standard formulae.

Phenotypic ($r_{p}$) and genetic ($r_{g}$) correlations between pairs of traits (x and y) were estimated using Equation (8) [41]:

(8) $$r=\frac{\sigma_{\left(xy\right)}}{\sqrt{\sigma_{\left(x\right)}^{2}\times \sigma_{\left(y\right)}^{2}}}$$

where $\sigma_{\left(xy\right)}$ denotes the covariance between traits x and y, and $\sigma_{\left(x\right)}^{2}$ and $\sigma_{\left(y\right)}^{2}$ are their respective variances.

#### 4.4.3. Sensitivity Analysis for Stem Form

To validate the genetic parameter estimates for stem form (SF) under conditions of low additive genetic variance and a sparse, skewed ordinal distribution, a Bayesian threshold mixed model was fitted using the MCMCglmm package in R [47]. The nine original SF scores were collapsed into three ordered categories (Low: 1–5; Medium: 6–7; High: 8–9), as scores 1–3 were sparse (n = 26). The model assumes a latent continuous liability underlying the observed ordinal scores, linked via a probit function, with the residual variance on the liability scale fixed at 1. Bivariate threshold–linear models, with family and family × replicate interaction included as random effects under an unstructured (us) covariance structure, estimated the genetic correlations between SF and each of the four primary selection traits (H, DBH, V, and CW). MCMC chains were run for 800,000 iterations with a burn-in of 200,000 and a thinning interval of 300; convergence was assessed using effective sample size (threshold > 1000) and Geweke’s diagnostic (threshold |z| < 2). This sensitivity analysis was conducted to verify the robustness of the primary LMM-based estimates and to provide a methodologically appropriate treatment of genetic correlations involving an ordinal trait [48,49].

#### 4.4.4. Selection Strategy and Genetic Gain

Given the low heritability and unstable genetic correlations estimated for SF, selection was performed using stem volume as the primary breeding objective and SF as a phenotypic culling criterion, corresponding to the independent culling levels approach [50,51] rather than a multi-trait index. At Stage 1, families were ranked by their volume BLUPs from the family model (3), and the 10 families with the highest values were selected. At Stage 2, individual volume BLUPs from the animal model (4) were used to identify elite trees from the top 10 families and from the remaining families with above-average volume BLUPs, retaining only individuals with SF ≥ 7. To maintain genetic diversity, up to two individuals were kept per top-10 family and up to one per other superior family.

Genetic gain (ΔG) for volume was calculated using Equation (9) as the difference between the mean breeding value of the selected cohort and that of the whole population.

(9) $$\Delta G=\frac{1}{m}\sum_{i=1}^{m}{\hat{a}}_{iS}-\frac{1}{n}\sum_{i}^{n}{\hat{a}}_{iALL}$$

where m is the number of selected individuals (or families) and n is the total number of individuals (or families) in the base population (i.e., all measured trees across the 113 families).

## 5. Conclusions

This study provides the first BLUP-based genetic evaluation of an 11-year-old Simao pine progeny trial comprising 113 open-pollinated half-sib families, drawn from plus-tree selections across the natural and planted distribution of the species in southern Yunnan. Family-mean heritabilities differed across the three growth traits, with tree height at 0.14, DBH at 0.29, and stem volume at 0.24, and the corresponding individual narrow-sense heritabilities were 0.08, 0.12, and 0.11. Stem volume showed the highest additive genetic coefficient of variation (GCV_A_ = 18.85%), supporting its use as the primary selection target. Volume-based BLUP selection delivered predicted genetic gains of 14.35% at the family level and 15.01% at the individual level. Stem form proved to be under weak additive genetic control, and a sensitivity analysis using a Bayesian threshold model demonstrated that anomalous LMM-based correlation estimates for this trait were attributable to insufficient additive variance rather than to true biological associations. Comparison of phenotypic and BLUP rankings across the five seed sources revealed that source-level mean performance was an unreliable predictor of breeding value rank, with Jinggu rather than the phenotypically superior Yunjing source dominating the top breeding-value families. Ten superior families and 35 elite individuals were identified through volume-based BLUP selection, with stem form applied as a phenotypic threshold for candidates intended for vegetative propagation. The selected materials provide an empirical foundation for the second cycle of Simao pine improvement and for clonal deployment in operational plantations. Future work should focus on multi-environment progeny trials to estimate genotype-by-environment interactions, on re-evaluation of the present trial closer to mid-rotation age to refine heritability and gain projections, and on the integration of wood quality traits into the breeding objective for subsequent selection cycles.

## Figures and Tables

**Table 1:** Characteristic and variance analysis of growth and qualitative traits of *P. kesiya* var. *langbianensis*.

Traits	Means ± SE		Family Means Range	PCV	F Value		
	Family	CK			Block	Family	Block × Family
H/m	13.29 ± 2.57	12.47 ± 2.43	10.86–15.61	16.21	528.57 **	4.13 **	3.56 **
DBH/cm	15.06 ± 3.55	14.96 ± 2.43	12.14–18.07	21.39	155.82 **	2.33 **	1.65 **
V/m^3^	0.135 ± 0.073	0.130 ± 0.061	0.080–0.207	47.56	241.34 **	2.70 **	2.02 **
HCB/m	7.48 ± 2.03	7.37 ± 2.04	0.5–19.5	22.72	422.80 **	3.95 **	4.49 **
CW/m	2.73 ± 0.60	2.67 ± 0.36	2.37–3.31	20.23	74.87 **	2.93 **	2.55 **
SF (score)	7.20 ± 1.30	7.28 ± 1.03	6.75–7.96	18.06	11.21 **	1.66 **	1.44 **

Note: Means ± SE; F-statistics for SF were computed on Blom-transformed values; descriptive statistics are reported on the original scale. ** = *p* < 0.01. CK refers to the commercial check lot planted as a reference in the trial (20 trees, four five-tree plots distributed across the four blocks). The CK was not included in the variance component analyses for the 113 half-sib families; its phenotypic means are reported here for reference purposes.

**Table 2:** Variance components and genetic parameters for growth and stem form traits at 11 years.

Trait	$\sigma_{f}^{2}$	$\sigma_{fb}^{2}$	$\sigma_{\varepsilon }^{2}$	$h_{f}^{2}$	$h_{s}^{2}$	GCV_A_
H (m)	0.082	1.466	2.704	0.14 ± 0.13	0.08 ± 0.02	9.02
DBH (cm)	0.314	1.107	8.909	0.29 ± 0.10	0.12 ± 0.01	9.84
V (m^3^)	0.0001	0.0006	0.0034	0.24 ± 0.11	0.11 ± 0.01	18.85
HCB (m)	0.000	1.221	1.729	0.00 ± 0.16	0.00 ± 0.09	0.00
CW (m)	0.005	0.080	0.250	0.12 ± 0.13	0.05 ± 0.02	1.71
SF (score)	0.009	0.052	0.755	0.09 ± 0.08	0.04 ± 0.03	-

Note: $\sigma_{f}^{2}$, family variance; $\sigma_{fb}^{2}$, family-by-block interaction variance; $\sigma_{\varepsilon }^{2}$, residual variance; $h_{f}^{2}$, family mean heritability; $h_{s}^{2}$, narrow-sense individual heritability; SE, standard error; GCV_A_, genetic coefficient of variation. Genetic parameters for stem form were estimated based on Blom-transformed data.

**Table 3:** Estimated phenotypic and breeding values for the top 10 selected families.

Family	Seed Source	No. of Individuals	Stem Volume (V m^3^)		Rank
			PV	BV	
15	1	20	0.0988	0.0290	1
19	1	20	0.0976	0.0267	2
30	1	20	0.0949	0.0212	3
16	1	25	0.0947	0.0209	4
17	1	20	0.0939	0.0192	5
89	1	20	0.0928	0.0171	6
40	1	25	0.0923	0.0160	7
87	1	20	0.0917	0.0149	8
193	2	20	0.0915	0.0143	9
145	1	20	0.0914	0.0142	10
Selected Mean			0.0940	0.0194	
Population Mean			0.0843	0.0000	
Genetic Gain (%)			14.35		

Note: PV, predicted phenotypic value estimated from the mixed linear model; BV, estimated breeding value (BLUP) derived as twice the general combining ability effect; Genetic gain (%) was calculated as the mean BV of the selected cohort divided by the population mean phenotypic value × 100. Seed sources: see Table 6 for details.

**Table 4:** Estimated genetic and observed values of the 35 selected elite individuals.

Rank	Individual ID	Family	Seed Source	Observed Value (V m^3^)	Stem Volume (V m^3^)		Stem Form
					PV	BV	
1	15_2_27	15	1	0.3383	0.1139	0.0296	7
2	89_4_1	89	1	0.3856	0.1105	0.0262	9
3	19_4_8	19	1	0.3383	0.1103	0.0260	8
4	193_2_35	193	2	0.3638	0.1102	0.0259	9
5	15_4_29	15	1	0.2901	0.1096	0.0253	7
6	30_3_5	30	1	0.3359	0.1094	0.0251	9
7	95_2_28	95	1	0.3741	0.1090	0.0247	7
8	19_4_4	19	1	0.3197	0.1084	0.0241	8
9	16_2_32	16	1	0.3158	0.1077	0.0234	8
10	17_2_48	17	1	0.3288	0.1076	0.0233	8
11	89_3_9	89	1	0.3358	0.1072	0.0229	9
12	144_4_6	144	3	0.3491	0.1069	0.0226	8
13	193_1_44	193	2	0.2664	0.1067	0.0224	8
14	40_4_10	40	1	0.3503	0.1065	0.0222	9
15	16_2_33	16	1	0.2962	0.1057	0.0214	9
16	30_2_46	30	1	0.2706	0.1055	0.0212	8
17	191_3_28	191	2	0.3605	0.1051	0.0208	8
18	50_3_1	50	1	0.3610	0.1047	0.0204	8
19	55_4_28	55	1	0.3380	0.1039	0.0196	8
20	156_2_31	156	4	0.3125	0.1038	0.0195	8
21	184_2_31	184	2	0.2881	0.1035	0.0192	9
22	87_3_5	87	1	0.3097	0.1026	0.0184	9
23	14_3_12	14	1	0.3281	0.1023	0.0180	8
24	143_3_2	143	3	0.3331	0.1021	0.0178	8
25	110_3_46	110	5	0.3176	0.1019	0.0176	7
26	147_1_27	147	1	0.2611	0.1017	0.0174	9
27	17_2_49	17	1	0.2709	0.1017	0.0174	7
28	18_2_43	18	1	0.2697	0.1014	0.0171	7
29	67_3_3	67	1	0.3093	0.1008	0.0165	9
30	179_3_24	179	4	0.2795	0.0997	0.0154	9
31	33_2_19	33	1	0.2766	0.0993	0.0150	8
32	145_4_2	145	1	0.2547	0.0989	0.0146	8
33	40_3_12	40	1	0.2588	0.0986	0.0143	9
34	145_3_1	145	1	0.2372	0.0981	0.0138	7
35	87_3_2	87	1	0.2192	0.0934	0.0091	8
Selected Mean (*n* = 35)				0.3039	0.1044	0.0203	
Population Mean(*n* = 2201)				0.1352	0.0843	0.0000	
Genetic Gain(%)				15.01			

Note: Individual No., composite identifier indicating family, block, and sequential tree number within the subplot. Selection limit: ≤2 individuals from each top-10 family, ≤1 individual for all other families. PV, predicted phenotypic value estimated from the mixed linear model; BV, estimated breeding value (BLUP) derived as twice the general combining ability effect; Genetic gain (%) was calculated as the mean BV of the selected cohort divided by the population mean phenotypic value × 100.

**Table 5:** Physical and chemical properties of the soil at the trial site.

Property	Mean ± SE	Range
pH (H_2_O, 1:2.5)	4.92 ± 0.09	4.67–5.18
Soil organic carbon (g kg^−1^)	6.12 ± 0.84	4.68–7.4
Total nitrogen (g kg^−1^)	0.62 ± 0.02	0.59–0.67
C/N ratio	9.9 ± 0.24	7.8–11.6
Total phosphorus (g kg^−1^)	0.19 ± 0.01	0.18–0.20
Total potassium (g kg^−1^)	16.19 ± 2.00	11.36–23.19
Available nitrogen (mg kg^−1^)	30.96 ± 2.65	25.1–40.4
Available phosphorus (mg kg^−1^)	0.53 ± 0.08	0.37–0.84
Available potassium (mg kg^−1^)	82.67 ± 8.87	54.85–109.7
Sand/Silt/Clay (%)	36.7/28.2/35.1	
Texture class (USDA)	Clay loam	

Values are means and ranges of five soil samples collected from the 0–60 cm layer within the trial site. Texture class follows the USDA system.

**Table 6:** Geographic origins and family details of *Pinus kesiya* var. *langbianensis* used in the progeny trial.

Seed Source	Latitude (°N)	Longitude (°E)	Family Number	Total Number of Families
1. Jinggu seed orchard	23°21′	100°33′	1, 5, 6, 7, 11, 12, 14, 15, 16, 17, 18, 19, 20, 22, 24, 25, 28, 30, 32, 33, 34, 35, 36, 37, 38, 40, 41, 42, 43, 44, 45, 46, 47, 48, 49, 50, 51, 52, 53, 54, 55, 56, 58, 61, 63, 64, 65, 66, 68, 85, 86, 87, 88, 89, 91, 94, 95, 103, 109, 120, 145, 146, 147, 150, 151, 152, 160	68
2. Yunjing seed orchard	23°37′	100°25′	181, 183, 184, 186, 188, 191, 193, 194, 199, 201	10
3. Jinggu resin base	23°15′	104°01′	104, 105, 106, 143, 144, 157, 163	7
4. Lianhua Town	22°52′	100°57′	108, 117, 122, 124, 126, 140, 154, 155, 156, 164, 165, 166, 167, 168, 169, 170, 171, 173, 178, 179	20
5. Nanping Town	22°45′	100°57′	107, 110, 111, 113, 123, 137, 138, 159	8

## Data Availability

The data presented in this study are available on request from the corresponding author. The data are not publicly available due to an ongoing breeding program.

## Supplementary Materials

The following supporting information can be downloaded at: https://www.mdpi.com/article/10.3390/plants15132035/s1, Table S1: Heritability estimates for stem form: Blom + LMM versus Bayesian threshold model; Table S2: Genetic correlations between stem form and growth traits from bivariate threshold–linear models; Table S3: MCMC convergence diagnostics for threshold models; Table S4: Sampling structure of the five seed sources at age 11.
