# Supplementary material for: Genetic Evaluation and Selection of Growth Traits of Pinus kesiya var. langbianensis Half-Sib Families
Source: Plants (Basel). 2026 Jun 30;15(13):2035. doi: 10.3390/plants15132035 (PMC13364300; doi:10.3390/plants15132035)
Supplement: Supplementary file 1 [file plants-15-02035-s001.zip › plants-4367501-supplementary.pdf]

## Supplementary Material

*Sensitivity analysis for stem form genetic parameters using Bayesian threshold mixed models*

**Table S1** Heritability estimates for stem form: Blom + LMM versus Bayesian threshold model.

| Method | $h_s^2$ mean | $h_s^2$ mode | HPD low | HPD high | Scale |
|---|---|---|---|---|---|
| Blom + LMM | 0.042 | 0.008 | 0.001 | 0.104 | Blom-transformed |
| Threshold (3-level) | 0.119 | 0.002 | 0.000 | 0.261 | Liability (probit) |

Note: The two methods operate on different scales (Blom-transformed observed scale vs. probit liability scale) and heritability values are not directly comparable. Both methods consistently indicate low additive genetic control of stem form (95% HPD intervals include or approach zero).

**Table S2** Genetic correlations between stem form and growth traits from bivariate threshold–linear models.

| Trait pair | $r_g$ mean | $r_g$ mode | HPD low | HPD high | Sig. | ASReml rg |
|---|---|---|---|---|---|---|
| Stem form × Height | 0.110 | 0.064 | –0.214 | 0.442 | ns | *NE* |
| Stem form × DBH | 0.093 | 0.120 | –0.221 | 0.437 | ns | — |
| Stem form × Volume | 0.109 | 0.152 | –0.206 | 0.420 | ns | — |
| Stem form × Crown width | –0.041 | –0.004 | –0.369 | 0.308 | ns | –0.865 |

Note: Stem form was modeled as ordinal (3-level) and growth traits as Gaussian. $r_g$ = genetic correlation on the liability scale. ns = not significant (95% HPD interval includes zero). NE = not estimable. — = estimate unreliable due to boundary variance. The anomalous ASReml estimate ($r_g$ = –0.865 for CW × SF) was an artifact of insufficient additive genetic variance rather than a true biological signal.

**Table S3** MCMC convergence diagnostics for threshold models.

| Model | ESS | ∣Geweke z∣ | Converged | Residual var. (SF) |
|---|---|---|---|---|
| Univariate threshold | 2000.0 | 0.330 | Yes | Fixed = 1 |
| Bivariate: SF × Height | 2000.0 | 1.583 | Yes | 1.000 (OK) |
| Bivariate: SF × DBH | 2106.4 | 0.350 | Yes | 1.000 (OK) |
| Bivariate: SF × Volume | 2000.0 | 1.765 | Yes | 1.000 (OK) |
| Bivariate: SF × Crown width | 2000.0 | 0.585 | Yes | 1.000 (OK) |

Note: ESS = effective sample size (target > 1,000). ∣Geweke z∣ = absolute Geweke convergence diagnostic (target < 2). SF = stem form. The residual variance for the ordinal trait was fixed at 1 on the probit liability scale. All models achieved satisfactory convergence.

*Sampling structure of the five seed sources at age 11*

**Table S4.** Sampling structure of the five seed sources at age 11.

| Seed source | No. of family | Total trees measured | Mean trees per family ± SD | Range |
|---|---|---|---|---|
| 1. Jinggu seed orchard | 68 | 1342 | 19.7 ± 4.3 | 15 - 34 |
| 2. Yunjing seed orchard | 10 | 178 | 17.8 ± 2.2 | 15 - 22 |

| | | | | |
|---|---|---|---|---|
| 3. Jinggu resin base | 7 | 135 | 19.3 ± 2.4 | 16 - 24 |
| 4. Lianhua Town | 20 | 382 | 19.1 ± 2.6 | 16 - 25 |
| 5. Nanping Town | 8 | 164 | 20.5 ± 4.5 | 15 - 34 |
| Total | 113 | 2201 | 19.6 ± 3.8 | 15 - 34 |

Note: All values refer to the number of surviving trees measured at age 11 (year 2024). Initial planting numbers per family ranged from 20 to 35 trees, reflecting the design of four five-tree plots per family (one per block) plus one or more additional five-tree plots for some families due to higher seedling availability during the 2013 planting. Natural mortality during the 11-year observation period resulted in surviving sample sizes per family ranging from 15 to 34 trees.
